# Shedding Light on the Cell Biology of Platelet-Derived Extracellular Vesicles and Their Biomedical Applications

**DOI:** 10.3390/life13061403

**Published:** 2023-06-16

**Authors:** Preeti Kumari Chaudhary, Sanggu Kim, Soochong Kim

**Affiliations:** Laboratory of Veterinary Pathology and Platelet Signaling, College of Veterinary Medicine, Chungbuk National University, Cheongju 28644, Republic of Korea; chaudharypreety11@gmail.com (P.K.C.); tkdrnfld@naver.com (S.K.)

**Keywords:** platelets, PEVs, hemostasis, inflammation, angiogenesis, wound healing, carcinogenesis, therapy

## Abstract

EVs are membranous subcellular structures originating from various cells, including platelets which consist of biomolecules that can modify the target cell’s pathophysiological functions including inflammation, cell communication, coagulation, and metastasis. EVs, which are known to allow the transmission of a wide range of molecules between cells, are gaining popularity in the fields of subcellular treatment, regenerative medicine, and drug delivery. PEVs are the most abundant EVs in circulation, being produced by platelet activation, and are considered to have a significant role in coagulation. PEV cargo is extremely diverse, containing lipids, proteins, nucleic acids, and organelles depending on the condition that induced their release and can regulate a wide range of biological activities. PEVs, unlike platelets, can overcome tissue barriers, allowing platelet-derived contents to be transferred to target cells and organs that platelets cannot reach. Their isolation, characterization, and therapeutic efficacy, on the other hand, are poorly understood. This review summarizes the technical elements of PEV isolation and characterization methods as well as the pathophysiological role of PEVs, including therapeutic potential and translational possibility in diverse disciplines.

## 1. Introduction

EVs are membranous subcellular structures originating from various cells, including platelets through a wide range of biomechanism [1]. EVs are divided into subpopulations based on morphology, size, content, cellular origin, and the functions they perform [2]. The EVs can bundle active cargo such as proteins, nucleic acids, and lipids and convey it to a recipient cell, whether close or far away, and thus, they can modify the destination cell’s pathophysiological functions including inflammation, cell communication, coagulation, and metastasis in the process [3]. EVs have enormous promise for the advancement of innovative biological treatments. Recently, EVs are being engineered as a disease-modifying biotherapy for age-related degeneration and as medication delivery vehicles for cancer, immunological, and inflammatory illnesses.

The most common type of EVs in circulation are PEVs, which are released upon activation of platelets by various factors [4]. PEVs have capabilities comparable to platelets and are thus thought to have an impact on a variety of biological processes such as coagulation, wound healing, and inflammation. PEVs have been shown to stimulate cellular differentiation, hence improving musculoskeletal or neurological regeneration. Unlike platelets, PEVs can pass across tissue barriers, extending their capabilities outside of the blood [5]. PEVs do not have legal and safety issues, can be obtained as a byproduct from whole-blood donations, bring no concerns regarding contamination or immunological reactions, and are unable to multiply because they lack a functioning nucleus. Therefore, using PEVs instead can have the desirable advantage of boosting the benefits of their clinical application. However, PEVs are poorly understood in terms of standardization, heterogeneity, repeatability, and storage conditions. It is unknown how PEVs package their machinery, transport it to other cells, and communicate between the cells in order to alter the pathophysiology of the target cells.

In this review, we summarize the technical features of PEV isolation and characterization approaches including the pathophysiological role of PEVs. In addition, this review will also look at the advantages and limitations of therapeutic applications of PEVs to grasp the fundamental needs for their clinical translation.

## 2. Extracellular Vesicles

EVs are lipid bilayer-delimited particles that are generated by nearly all types of cells in a normal manner but are unable to proliferate like cells. EVs are a tool for intercellular communication, facilitating the interchange of a wide variety of chemicals between nearby or far-away cells. EVs usually contain lipids, nucleic acids, and proteins, particularly those connected to the cell membrane, the cytosol, and those involved in lipid metabolism [6,7]. The diversity of cell types and functional states, as well as the various biogenetic pathways, all contribute to the variability of EVs. Exosomes, microvesicles, and apoptotic bodies are the three primary subtypes of EVs, distinguished by their biogenesis, release mechanisms, size, composition, and function [8]. As a brief explanation, exosomes are encased in a single outer membrane, typically ranging from 30 to 150 nm, released by all types of the cell through the endosomal pathway, and present in various kinds of body fluids [6,7]. Exosomal vesicles specifically originate by inward budding of early endosomes’ limiting membranes, which develop into multivesicular bodies (MVBs) in the process [9]. Exosomes are engaged in the cell’s endocytic and material trafficking processes, playing a part in protein sorting, recycling, storage, transport, and release, specifically [10]. Alix, TSG101, HSC70, and HSP90 are expected to be expressed by exosomes and can be used as “exosomal marker proteins” [11,12]. Exosomes frequently contain the tetraspanin proteins including CD63, CD9, and CD81. Exosomes have a role in cell-to-cell communication, cell maintenance, tumor progression, and cellular waste management, and they behave as antigen-presenting vesicles and promote immunological responses [13].

Microvesicles are EVs that develop from the cell membrane by directly outward budding, or pinching, and typically have diameters between 100 nm and 1000 nm. Microvesicles mostly contain cytosolic and cell membrane-associated proteins, such as tetraspanins. Integrins, heat shock proteins, cytoskeletal proteins, and proteins with post-translational modifications including glycosylation and phosphorylation are other proteins that are frequently found in microvesicles [14]. Initially, it was believed that microvesicles were a cellular dumping or maintenance process, similarly to exosomes, by which the cell would get rid of waste [9]. However, microvesicles are now recognized to play a role in cell–cell communication between nearby and distant cells. Likewise, dying cells discharge apoptotic bodies into the extracellular environment. According to reports, they can be as little as 50 nm or as large as 5000 nm in diameter, with most apoptotic bodies being on the larger side [15]. During cell contraction, increasing hydrostatic pressure causes the cell membrane to separate from the cytoskeleton, resulting in the generation of these apoptotic bodies [16]. Apoptotic bodies, as opposed to exosomes and microvesicles, include intact organelles, chromatin, and modest levels of glycosylated proteins [16]. So, it makes sense to anticipate seeing larger quantities of proteins associated with the nucleus, mitochondria, Golgi apparatus, and endoplasmic reticulum (such as histones), among other structures.

## 3. Origin of PEVs

Platelets are anucleated, discoid cells that originate from megakaryocytes in the bone marrow and circulate in the bloodstream with a physiological count of 150,000 to 450,000 platelets/µL of blood and a lifespan of 8–10 days. The resting platelet consists of alpha granules, dense granules, lysosomal granules, and glycogen granules, which contain various kinds of proteins, growth factors, angiogenic factors, chemokines, immune mediators, etc. that are involved in various pathophysiological activities of the platelet (Table 1) [17]. The activation of platelets leads to the development of hemostasis and thrombosis. Upon vascular injury, von Willebrand Factor (vWF) and collagen become exposed to the extracellular matrix which binds with their respective receptors present in the platelet and activates the platelet. The activated platelet releases adenosine diphosphate (ADP) and generates thromboxane A_2_, which further recruits the circulating platelet in the bloodstream, activates them, and forms the hemostatic plug by converting fibrinogen to fibrin in the presence of thrombin.

Under normal circumstances, the circulating microvesicles found in the plasma come mostly from megakaryocytes. However, in pathological conditions, microvesicles are produced by activated platelets. Upon activation of platelets by a variety of agonists, platelets readily generate EVs as well (cellular plasma membrane (microvesicles) or endosomal compartment (exosomes)) that remain circulating in the bloodstream. Chargaff and West initially documented PEVs as coagulant lipoproteins that were separated from platelets by differential centrifugation [18]. Shortly after, electron microscopic analyses of α-granule release from platelets also imaged small vesicles being released, which were referred to as exosomes [19,20]. Data from various electron microscopy demonstrated that depending on the type of agonist stimulation, there are two types of PEVs released: small vesicles (exosomes) with a diameter of ~40 to 100 nm that expose CD63 and are undetectable by flow cytometry, and larger vesicles (microvesicles) with a diameter of 100 to 1000 nm that expose annexin-V and express αIIb-β3 and β1, GP1bα, and P-selectin, which enables Factor X and prothrombin [14,21]. It is widely acknowledged that megakaryocytes and platelets are the main sources of EVs in blood circulation [22,23]. Another research demonstrated the first proof that PEVs may have both pro- and anti-coagulatory effects, even though PEVs had previously only been linked to procoagulant activity [24]. Now, PEV cargo is considered to be highly diverse consisting of proteins, growth factors, nucleic acids, and organelles that are present in the platelet itself (as shown in Table 1) and are engaged in diverse biological activities in different cell types. PEVs can infiltrate into various organs and tissues where they contribute to more distant cellular communication. This makes it possible to deliver platelet-derived material to cells and organs that platelets cannot reach.

**Table 1:** Various chemical modulators present in platelets.

Location	Type	Chemical Modulators	References
α-granules	Adhesive proteins	P-selectin Fibrinogen Von Willebrand factor Fibronectin Thrombospondin-1 Thrombospondin-2 Laminin-8 Vitronectin	[25,26,27,28,29,30,31]
	Growth factors	EGF IGF-1 HGF TGF-β PDGF	[32,33,34,35,36]
	Angiogenic factors	VEGF PDGF FGF	[37,38,39]
	Chemokines	CXCL1/2/5/6/7/8/12 CCL2/3/5/7 (RANTES) IL1β CD40L Proteases	[40,41,42,43,44,45,46,47,48,49,50]
	Coagulation factors	Factor V Protein S Factor XI Factor XIII Kininogens Plasminogen	[51,52,53,54,55,56]
	Integral membrane proteins	Integrin αIIbβ3 GPIba-IX-V GPVI TLT-1 P-selectin	[25,57,58,59,60]
	Immune mediators	Complement C3/C4 precursor Factor D/H C1 inhibitor Immunoglobulins	[61,62,63,64,65]
	Protease inhibitors	α2-antiplasmin PAI-1 α2-antitrypsin α2-macroglobulin TFPI C1-inhibitor	[64,66,67,68,69,70]
	Proteoglycans	MMP2, MMP9	[71]
Dense granules	Amines	Serotonin Histamine	[72,73]
	Bivalent cations	Ca^2+^ Mg^2+^	
	Nucleotides Polyphosphates	ATP ADP GTP GDP	

EGF: epidermal growth factor; IGF-1: insulin-like growth factor 1; HGF: hepatocyte growth factor; TGF-β: transforming growth factor-β; PDGF: platelet-derived growth factor; VEGF: vascular-endothelial growth factor; FGF: fibroblast growth factor; PAI-1: plasminogen activator inhibitor-1; TFPI: tissue factor inhibitor; MMP: matrix metalloprotease.

## 4. Isolation and Detection of Platelet-Derived Extracellular Vesicles

While there are several PEV isolation techniques that have been invented, the lack of consistent and optimum techniques is a significant barrier to introducing exosomes in the clinical and experimental field. Most of the PEV isolation methods are divided into platelet isolation and PEV isolation. Platelets must be very pure at this time, so not only simple centrifugation methods but also a 10–17% iodixanol gradient or leukocyte reduction filtration by PVC-citrate storage bag are used to isolate completely pure platelets [23,74]. Additionally, PEVs can be isolated from platelet lysate [75,76], platelet derivatives, and plasma [77]. After that, platelets can be completely activated not only with platelet agonists including collagen, thrombin, collagen-related peptide (CRP), ADP, and thrombin receptor-activating peptide (TRAP)-6, but also with lipopolysaccharide (LPS), Ca^2+^ ionophore, and LPS-binding protein [23]. The most commonly known method is the centrifugation technique, which obtains PEVs from the supernatant from which platelets and cell debris have been removed through centrifugation. This technique can also separate various types of EVs such as microvesicles and exosomes by adding more steps of centrifugation. However, it has been known that high-speed centrifugation should be used with precaution because it can affect the concentration of EVs, their size, or their biochemical composition via the generation of EV aggregates [78]. In addition, gel-filtration via size-exclusion chromatography, immunoaffinity chromatography using disk with anti-human CD61 antibody [77], and/or anti-CD-41, CD63, CD9, and CD81 antibody-covered beads can be applied to further separate the desired PEVs [74,75,79,80]. Although it is not currently applied to PEV isolation, precipitation is known to be used as a technique for EV isolation and purification in several other cells [81]. Detailed PEV preparation conditions that can be applied are mentioned in Table 2.

## 5. Analysis and Detection of Platelet Extracellular Vesicles

After the preparation of the PEVs, several analyses are applied for confirmation and characterization of them. One of the most commonly used methods is nanoparticle tracking analysis (NTA). This method gives the position, vesicle concentration, and size of particles suspended in a fluid by detecting the light they scatter [85]. It can detect nanoparticles ranging from 10 nm to 2000 nm. However, particles smaller than 50 nm are not well identified, and contaminants in the sample such as protein aggregates and cell fragments can be detected. In this case, utilizing 0.02 µm filtration or immuno-labeling the particle can specify the target particles [86]. Dynamic light scattering (DLS), a method similar to NTA, estimates scattering intensity from bulk samples, unlike NTA. The main advantage of DLS is that it can measure particles ranging from 1 nm to 6 µm. However, purification is still required because the data can only be trusted if just there is the presence of one sort of particle [87]. Flow cytometry, a method of detecting, counting, and sorting single-file passage targets using a laser beam, is another approach often employed in EV analysis [88]. This approach has the advantage of determining the absolute number of particles, although it only detects particles over 200 nm and frequently detects many vesicles as one when concentrations are high (swarming effect) [89]. Furthermore, electron microscopes are utilized for PEV detection, and not only the size of the vesicle but also its exact form can be viewed through direct measurement, but structural damage may be occurred through high surface tension during evaporation of water [90]. There are many additional approaches, such as tunable resistive pulse sensing, atomic force microscopy, and mass spectrometry, but it is important to evaluate based on the purpose and size of the target.

## 6. PEVs in Health and Diseases

**Figure 1:**
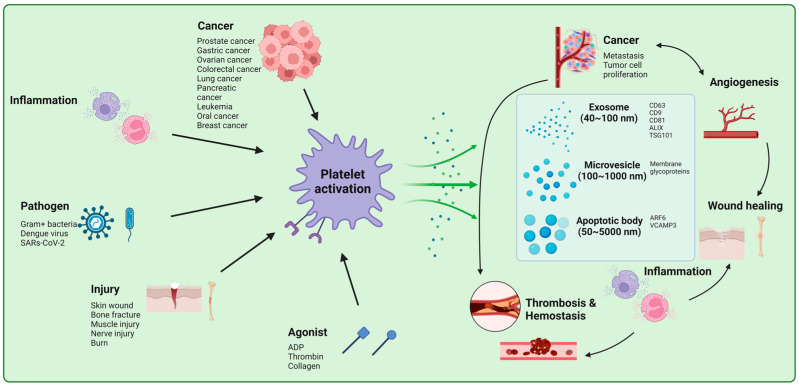
The effect of PEVs in diverse pathophysiological processes. Activation of platelet by various stimuli including cancer, inflammation, pathogens, and injury leads to the release of EVs: exosomes, microvesicles, and apoptotic body The EVs can bundle active cargo such as proteins, nucleic acids, and lipids and convey it to a recipient cell, whether close or far away; thus, they can modify the destination cell’s pathophysiological functions including inflammation, cell communication, angiogenesis, coagulation, and metastasis in the process. “Created with BioRender.com”.

PEVs have been associated with both noninfectious chronic inflammatory diseases (e.g., atherosclerosis, diabetes, coronary artery disease, and hypertension) and infectious diseases (e.g., influenza and COVID-19) as well as other pathophysiology. The diverse roles of PEVs in various fields are shown in Figure 1 and are summarized below: 

### 6.1. PEVs in Hemostasis, Coagulation, and Hemorrhagic Shock

Worldwide, trauma is responsible for more than 500,000 deaths each year, and severe hemorrhage leading to trauma-induced coagulopathy (TIC) characterizes the majority of these cases [91]. Platelet and plasma therapy have been shown to decrease hemorrhage-associated mortality in TIC patients [92]. One of the known primary activities assigned to platelet EVs is coagulation [4,18]. The surface of circulating PEVs has been shown to be 50–100-fold more procoagulant than activated platelets [93]. Negatively charged phosphatidylserine and tissue factor (TF) exposed to the surface of PEVs have been substantially attributed to their pro-coagulant activities. PEVs express binding sites for coagulation factors such as activated factor V and factor VIII as well as thrombin [93,94,95]. EV clearance was recently demonstrated to be aided by coating PS with lactadherin, which reduced coagulopathy and improved survival in a traumatic brain injury mouse model [96]. PEVs produce activated protein C, which is known to be involved coagulation process [24]. Following severe trauma, treatment with PEVs has been shown to enhance hemostasis, stop blood loss, and slow the development of hemorrhagic shock [85]. Since platelets cannot be kept and must be utilized within five days of withdrawal, PEV preparation clearly outperforms platelet transfusion in terms of storage. Investigating plasma proteins, blood cells, and the endothelium is important to have a thorough grasp of the hemodynamic physiology of PEVs. Taken together, although platelets and PEVs have numerous functional similarities, including a strong procoagulant capacity, PEVs may be a better option for hemostasis. However, before these encouraging results can be applied to clinical practice, more study is required to characterize the PEV isolates in greater detail. Investigating the interaction between proteins and cells present in the blood, and the endothelium is important to have a thorough grasp of hemodynamic physiology. By doing so, we can explore the dynamics of the biomarkers and have the opportunity to gauge the treatment’s immediate impact on coagulation.

### 6.2. PEVs in Immune Response and Inflammation

PEVs have a significant effect on the pathophysiology of the immune system by increasing in quantity or changing granule contents. PEV is released by platelet activation mediated by platelet agonists in a physiologic state and inflammation and/or infection in a pathologic state [97,98]. Various viruses trigger the release of PEV, of which dengue virus induces PEV release through c-type lectin (CLEC)-2 of the platelet [99]. Additionally, COVID-19 has recently been found to induce PEV release through CLEC-2 [100] and elevate PF4^+^ and HMGF1^+^ PEVs, which are elevated in sepsis [101,102]. It is known that several Gram-positive bacteria increase the release of EVs, but this has not been elucidated in PEVs [103]. Generated PEVs are involved in a variety of immune-related pathways, and they interact with mononuclear cells more than other inflammatory cells. PEVs can deposit inflammatory mediators such as CCL5 onto the endothelium surface, resulting in the recruitment of mononuclear cells during rolling [104]. Additionally, PEVs increased the adhesion of monocytes to endothelial cells by arachidonic acid and protein kinase C activation in a time- and dose-dependent manner [105]. In addition, PEVs bind to monocytes via p-selectin-p-selectin glycoprotein ligands-1, and this is sustained by phosphatidylserine binding, at which GPIbα transfers to the monocyte, which is recruited into the blood vessel and stimulate angiogenesis [106]. Additionally, PEVs have the ability to change the distribution of monocyte subsets towards intermediate CD14^+^ CD16^+^ monocytes with inflammatory properties [107]. PEVs also can significantly increase the expression of MMP 9, hydrogen peroxide, and pro-inflammatory factors, including C5a and tumor necrosis factor (TNF) [108]. PEV is also known to interact with several other inflammatory cells. Recently, PEVs have been known to interact with T-lymphocyte [109]. PEVs induced the differentiation of naive CD4^+^ T Cells into Foxp3^+^ regulatory T cells by TGF-β, and they induced immunosuppressive response by decreasing the release of IFNr, TNFα, and IL-6 [110]. Furthermore, unlike platelets, PEVs circulate in lymph and express MHC-1 to perform co-activation with lymphocytes via CD40L and OX40L [111]. However, the role of PEVs in T-lymphocyte, including proliferation, differentiation, and cytokine production, needs further research [109]. Additionally, it is known that PEVs can enhance inflammatory response by capturing and activating neutrophils and endothelial cells to promote interaction [112]. In particular, PEVs worsen the symptoms by activating neutrophils through heterocomplexes of TLR2 and CLEC5a [99], and they increase the activity of EV-TF [113]. PEVs eventually induce a number of diseases, including rheumatoid arthritis (RA) and systemic lupus erythematosus (SLE). PEVs in RA enter the lymphatic system and influence joint vascular leakage via the fibrinogen receptor αIIbβ3 and serotonin [114]. A high number of influxed microvesicles, the majority of which carried the platelet marker CD41a, were found in synovial fluid from an RA patient and stimulated monocyte adhesion to the endothelium, thus increasing the ICAM-1 in monocytes [115,116]. In addition, presence of intra-vesicular arachidonic acid in PEVs increases monocyte adhesion to endothelial cells, which further transfers the lipids and lipid metabolism in cells, eventually inducing atherosclerosis and inflammation [117]. Furthermore, PEVs promote inflammation through serotonin and IL-1 in SLE an autoimmune disease, as well as RA [118,119]. PEVs also can spread infection by delivering functional viral RNA from cell to cell in several viral infections [120,121]. As a result, PEVs are intimately linked to immunity, including release by immune-mediated disease, interaction with inflammatory cells, and expression of immune-mediated disease symptoms.

### 6.3. PEVs in Angiogenesis and Wound Healing

PEVs can indirectly increase angiogenesis in the inflammatory induction and the subsequent hypoxic condition in the vascular injury [122]. However, PEVs further induce angiogenesis through various mechanisms. PEVs are known to secrete various angiogenic growth factors including lipid growth factors (sphingosine-1-phosphate) [123] RANTES [124] and several growth factors (VEGF, FGF-2, bFBF, PDGF, TFG-beta, EGF, hybridoma growth factor, MMP-2, MMP-9) [123,125,126,127,128]. Increased VEGF, FGF-2, and lipid growth factors induce endothelial progenitor cells differentiation, endothelial proliferation, chemotaxis, tube formation, and stimulating resident mature endothelial cells via PTX-sensitive G protein, extracellular signal-regulated kinase, phosphoinositide 3-kinases, AKT, and Src kinase activation [123,125,126,127,129]. Furthermore, PEVs amplify the vaso-regenerative potential of endothelial progenitor cells (EPCs) and support the maintenance of vascular integrity after arterial injury through recruitment, migration, and differentiation via CXCR4 sensitization and by providing CD31, vWF, and lectin phenotype of EPCs [129,130]. PEVs also contain angiogenic microRNAs (miRNAs), including miR-320, miR-25, and miR-126. Among these, miR-126 has the ability to down-regulate vascular cell adhesion molecule-1 upon the VEGF, thereby contributing to endothelial migration and proliferation [131]. The angiogenic effect of PEV changes in several disease conditions. PEVs in patients with pulmonary arterial hypertension induced more transcription and translation of VEGF-A, and FGF, further promoting endothelial cell activation through escape lysosomal degradation [132]. In addition, the PEVs improve the process of revascularization in ischemic myocardium [125]. On the other hand, Nitric oxide (NO) and bacterial elements can trigger the PEV release and further induce caspase-3 activation and apoptosis of target endothelial cells through active ROS/RNS generation by NADPH oxidase and NO synthase via redox-signaling pathway type II in sepsis [133,134]. In addition, PEVs secreted from PM2.5 (fine dust)-exposed platelets significantly reduced the proliferation of vascular endothelium by changing miRNAs level, decreasing the effective angiogenic factors and increasing proinflammatory factors (ICAM-1, IL-6, and TNF-a), ROS level, and apoptosis (up-regulation of cytochrome-C, BAX, and cleaved caspase-3, and down-regulation of Bcl-2) [135]. As a result, PEVs can be an effective therapeutic target in a certain disease state, and they can also be a candidate for investigating delayed or augmented angiogenesis in numerous diseases with unknown angiogenesis mechanisms.

### 6.4. PEVs in Carcinogenesis

PEVs are influenced by tumor cells and, in turn, influence tumor cells by various mechanisms in different tumor cell types. It is known that PEVs are increased by several types of tumors including tumor including prostate cancer [136,137], gastric cancer [138], ovarian cancer [139], colorectal cancer [140,141,142], lung cancer [143], pancreatic cancer [141], acute lymphoblastic leukemia, oral squamous cell carcinoma [144], and breast cancer [145]. Increased PEVs raise the level of factors, such as VEGF, IL-6, RANTET, fibrinogen, and TNF-α, which increases metastasis and cancer grade [138,144], and eventually, this significantly reduces median survival time [136]. This increase in PEVs is especially pronounced in the presence of a large tumor, distant metastases, or invasiveness [145]. In addition, the increase in D-dimer may have come from an increase in PEVs, which promotes thrombosis [137,139,140]. All of these tumor-induced PEV alterations are also seen in proteomics. Increased HLA and PSMD2 levels in PEVs derived from colorectal cancer patients promote immune response, and an increase in HLA elevates platelet activity, resulting in accelerated carcinogenesis [142]. However, further research is needed to determine whether the increase in PEVs number is the result of chemotherapy [136,143,146,147].

On the other hand, PEVs also affect tumor cells. PEVs enhance tumor cell invasion by stimulating MMP-2 synthesis and secretion [148]. PEVs transfer CD41 to lung cancer and induce the phosphorylation of mitogen-activated protein kinase (MAPK) p42/44, serine/threonine kinases, and membrane type 1-matrix metalloproteinase (MT1-MMP), which stimulate proliferation, upregulate cyclin D2 expression, and increase trans-Matrigel chemo-invasion [149]. Furthermore, PEVs stimulate mRNA expression of angiogenic factors, including MMP-9, VEGF, IL-8, and HGF, which promote lung cancer metastasis. Additionally, miR-939 in PEVs increases the migration, proliferation, and expression of molecules associated with epithelial–mesenchymal transition in epithelial ovarian cancer mediated by sPLA_2_-IIa [150]. Tropomyosin 3 (TPM3) mRNA, which has been associated with metastasis in breast cancer, significantly increases the platelet of breast cancer patients, which transmit TPM3 mRNA to breast cancer through PEVs, giving breast cancer a migrative phenotype [151]. Moreover, PEVs aggregate breast cancer cells, bind and internalize to the breast cancer cell, and stimulate migration and invasion via phosphorylating p38 MAPK and myosin light chain [152]. PEVs from colorectal cancer patients accelerate metastasis by increasing EMT markers TWIST1 and VIM in the colorectal cancer cell line, as well as enhancing COX2 and TxA2 generation to promote cancer development [142]. In addition, colorectal cancer cell line acquired the capacity to produce 12-HETE from PEVs generated from platelet type 12-LOX, which is detected in adenoma or adenocarcinoma patients [153]. Furthermore, EMT genes expression are suppressed by 12-LOX inhibitors, suggesting that they can be utilized as a treatment for cancer. However, PEVs can suppress the growth of lung and colon carcinomas by miR-24 by inhibiting mitochondrial noncoding small nucleolar RNA mt-Nd2 and Snora75 [154]. PEVs also inhibit the expression of the EMT marker CDH1 [142]. Not only the characteristics of each tumor by PEVs but also the contradictory findings of PEVs on tumor growth should be thoroughly investigated in order to identify the mechanisms and efficient treatment of the tumor in the future.

## 7. Therapeutic Applications and Future Perspectives of PEVs

PEVs are bioproduct that have recently been used in regenerative medicine through their functions including inflammation, hemostasis, angiogenesis, and cell proliferation, and it is gaining attention dues to their efficiency more than platelets [155]. In addition, the research has not been thoroughly conducted, and thus, possibilities in various fields are expected. In breast cancer cell lines (MDA-MB-231, SKBR3, and BT474 but not MCF-7 cells), PEVs efficiently interact with all except MCF-7 [156]. Similarly, in rheumatic arthritis, platelet exosome displays antigens that are detected by rheumatic arthritis-specific autoantibodies [157]. In the case of a COVID-19 patient, proteins linked to cardiovascular disease and pro-thrombotic/endothelial damage factors were elevated in EVs from severe cases. However, in moderate cases, levels of TF, CD163, and EN-RAGE were lower compared to severe cases [157]. Thus, if proteomic and genomic profiling of PEVs is completed, it can serve as a biomarker. Furthermore, PEVs can bind with integrin and membrane glycoproteins such as GPIIbIIIa (CD41/CD61 or integrin αIIbβ3), GPIaIIa (CD49b/CD29), GPIba (CD42b), P-selectin (CD62P), platelet endothelial cell adhesion molecule-1 (CD31), and GP53, allowing us to employ it in drug delivery. Additionally, since cells inhibit apoptosis by exporting their intracellular caspase-3 through EVs to perform waste management, platelet homeostasis research can be undertaken by controlling PEV release [158]. Moreover, it has been known that cells secrete viral DNA and RNA via EV in a viral infection state, and thus, it will be available as an identifying mechanism for spreading and as a new virucidal target [159,160]. In addition, PEVs are taken up by cancer cells, causing them to produce corresponding substances by transferring molecules (as discussed earlier) [153]. Beyond just drug delivery, this mechanism can provide a new approach to preventing or improving various pathological conditions. However, there are many hurdles to using these PEVs in clinical practice. PEVs derived from optimized preparation from blood without blood-related infectious diseases should be treated with appropriate treatment methods and should be kept for a long time without degradation. Additionally, we should know exactly in which diseases PEVs should not be utilized.

## Figures and Tables

**Table 2:** Various PEV preparation conditions used to date [23,75,76,77,80,82,83,84].

Method of Isolation	Approaches	Advantages	Disadvantages
**Platelet activation**	**Activation for 30 min** - Thrombin 1 U/mL - Collagen 10 µg/mL - CRP-XL 1 µg/mL - ADP 60 µM - TRAP-6 10 µM - Thrombin 1 U/mL + collagen 10 µg/mL - Ca^2+^ ionophore 10 µM **Activation for 3 h** - LPS 100 ng/mL - LBP 100 ng/mL - CD14 100 ng/mL **Activation by CaCl_2_**	- Characteristically different types of PEVs can be produced - Enhances PEVs release	- Lower procoagulant activity - Expensive
**Centrifugation**	**PEV preparation** - 800–5000× *g* for 5 min–30 min **Purification** - 20,000× *g* for 60 min **Microvesicle pellet preparation** - 2500–12,000× *g* for 15 min–60 min **Exosome pellet preparation** - 20,000–120,000× *g* 40 min–18 h	- Cost efficient - Pure preparation	- Low reproducibility - Possibility of exosomes damage
**Membrane filtration**	**PEV preparation** - 0.2 µm pore membrane filtration **PEV purification** - 0.8 µm pore membrane filtration	- Simple procedure - Process many samples at the same time - Pure preparation	- Deformation of vesicles (less exosomal proteins)
**Gel filtration (size exclusion chromatography)**	**Further isolation** - Isolating 0.5 mL of 26 fraction, harvest fraction 9–12 - Isolating 24–30 fraction	- High reproducibility - Pure preparation - Preserves vesicle integrity - Prevent PEV aggregation	- Need specialized equipment - Expensive
**Immunoaffinity chromatography**	**Further isolation** - Filtering sample with the disk with anti-human CD61 antibody at a flow rate of 0.5 mL/min repeated five times.	- Fast and easy - Enrichment of hundred to thousand-fold	- Expensive
**Iodixanol density gradient**	**Further isolation** - Collect the band from the 30% and 10% interface	- Pure preparations without viral particles	- Sample loss - Unable to separate large particles with similar sedimentation rates
**Immuno-bead capturing**	**Further isolation** - Incubate sample with Anti-CD63, CD9, and CD81 antibody covered beads	- High reproducibility - Pure preparation	- Not suitable for large-volume samples

## Data Availability

Data is contained within the article.

## Abbreviations

EV	Extracellular vesicle
PEV	Platelet-derived extracellular vesicle
vWF	Von Willebrand factor
ADP	Adenosine diphosphate
EGF	Epidermal growth factor
IGF-1	Insulin-like growth factor-1
HGF	Hepatocyte growth factor
TGF-β	Transforming growth factor-β
PDGF	Platelet-derived growth factor
VEGF	Vascular-endothelium growth factor
FGF	Fibroblast growth factor
PAI-1	Plasminogen activator inhibitor-1
TFPI	Tissue factor inhibitor
MMP	Matrix metalloprotease
CRP	Collagen-related protein
TRAP	Thrombin receptor-activating protein
LPS	Lipopolysaccharide
NTA	Nanoparticle Tracking Analysis
TIC	Trauma-induced coagulopathy
TF	Tissue factor
CLEC	C-type lectin
RA	Rheumatoid arthritis
SLE	Systematic lupus erythematosus
EPC	Endothelial progenitor cell
miRNAs	Micro RNAs
NO	Nitric Oxide
MAPK	Mitogen-activated kinase
